# Analysis of Gene Expression in Induced Pluripotent Stem Cell-Derived Human Neurons Exposed to Botulinum Neurotoxin A Subtype 1 and a Type A Atoxic Derivative

**DOI:** 10.1371/journal.pone.0111238

**Published:** 2014-10-22

**Authors:** Jacob M. Scherf, Xiaoyang Serene Hu, William H. Tepp, Konstantin Ichtchenko, Eric A. Johnson, Sabine Pellett

**Affiliations:** 1 Department of Bacteriology, University of Wisconsin-Madison, Madison, Wisconsin, United States of America; 2 Department of Pharmacology, New York University School of Medicine, New York, New York, United States of America; Institute Pasteur, France

## Abstract

Botulinum neurotoxin type A1 (BoNT/A1) is a potent protein toxin responsible for the potentially fatal human illness botulism. Notwithstanding, the long-lasting flaccid muscle paralysis caused by BoNT/A has led to its utility as a powerful and versatile bio-pharmaceutical. The flaccid paralysis is due to specific cleavage of neuronal SNAREs by BoNTs. However, actions of BoNTs on intoxicated neurons besides the cleavage of SNAREs have not been studied in detail. In this study we investigated by microarray analysis the effects of BoNT/A and a catalytically inactive derivative (BoNT/A *ad*) on the transcriptome of human induced pluripotent stem cell (hiPSC)-derived neurons at 2 days and 2 weeks after exposure. While there were only minor changes in expression levels at 2 days post exposure, at 2 weeks post exposure 492 genes were differentially expressed more than 2-fold in BoNT/A1-exposed cells when compared to non-exposed populations, and 682 genes were differentially expressed in BoNT/A *ad*-exposed cells. The vast majority of genes were similarly regulated in BoNT/A1 and BoNT/A ad-exposed neurons, and the few genes differentially regulated between BoNT/A1 and BoNT/A *ad*-exposed neurons were differentially expressed less than 3.5 fold. These data indicate a similar response of neurons to BoNT/A1 and BoNT/A *ad* exposure. The most highly regulated genes in cells exposed to either BoNT/A1 or BoNT/A *ad* are involved in neurite outgrowth and calcium channel sensitization.

## Introduction

Botulinum neurotoxins (BoNTs), the causal agents of botulism, are considered the most potent toxins to humankind [1], [2]. Of the seven currently known serotypes, BoNT/A has been shown to be particularly potent in humans; an ingested dose of 1–2 µg/kg body weight and an intravenous dose of 1–2 ng/kg is estimated to be fatal unless prompt medical treatment is received [3]–. BoNTs prevent neurotransmitter release by targeting the neuromuscular junction (NMJ), leading to flaccid muscular paralysis, with death usually caused by respiratory paralysis [2]. The severe effects of BoNTs are also due to the particularly long persistence of symptoms, lasting several months and up to 1 year in the case of BoNT/A, after which time the patients usually recover completely [2]. Despite the potentially fatal nature of BoNTs, the potency, duration, and reversible action of these neuroparalytic toxins has led to extensive applications in medicine. BoNT/A1, and to a lesser extend BoNT/B1, is now widely used as a unique and important bio-pharmaceutical to treat a variety of neuromuscular disorders, for cosmetic purposes, and to treat a variety of nervous-system-related disorders, such as migraine headaches, muscle spasticity, and various types of dystonia [6]–[8].

BoNTs specifically enter neuronal cells via receptor-mediated cell entry [9]. After binding to polysialogangliosides, BoNTs associate with synaptic vesicle proteins (in the case of BoNT/A SV2C) and are endocytosed. Once inside the endocytic vesicle, acidification of the vesicle lumen leads to a conformational change in the BoNT protein, which ultimately leads to translocation of the light chain (LC) into the cell cytosol [10], [11]. The disulfide bridge connecting the heavy chain (HC) and LC is reduced in the cell's cytosol [10]–[12], and the enzymatically active LC is released. BoNTs interrupt signal transduction by specifically cleaving a soluble N-ethylmaleimide-sensitive-factor attachment protein receptor (SNARE) protein; in the case of BoNT/A, the t-SNARE synaptosomal-associated protein 25 (SNAP-25) [13].

Upon entering the neuronal cell and cleaving SNAP-25, the toxin's LC remains active in the cytosol and cleaves newly synthesized SNAP-25, preventing recovery for an extended period of time [14]–[16]. Recovery is slow, and despite widespread use of BoNT/A as a therapeutic, cellular processes activated inside intoxicated neurons have largely remained unstudied in detail. Due to the specific neuronal cell entry, BoNT has been considered as a potential vehicle for delivering beneficial agents directly to neuronal cells [17]. Owing to the complicated and only partially understood mechanism of cell entry by BoNTs, a recently developed BoNT based specific neuronal delivery vehicle was designed based on a full length atoxic derivative of BoNT [18]. This derivative, BoNT/A *ad*, has been shown to be catalytically inactive at pM concentrations as used here, and has been proposed as a candidate for a neuron-targeting delivery mechanism [18], [19]. Although the intoxication pathway of BoNT/A *ad* has been investigated using *in situ* and neuronal cell models [19], the effect of the catalytically inactive toxin derivative on the human neuronal transcriptome remains unknown, especially compared to the effect of catalytically active BoNT/A. In addition, the effect of physiologically relevant doses of BoNT/A1, the BoNT serotype most commonly used for medical applications today, on the human neuronal transcriptome has not been investigated either.

The few functional studies in neurons exposed to BoNT/A1 have shown that there is post-intoxication neurite outgrowth in various human and animal models [20]–[25]. Other studies have investigated the effect of BoNTs on human epithelial cells and neuronal cell lines [26], as well as human skeletal muscle [27] and keloid fibroblasts [28]. These studies observed differential regulations of a wide range of genes involved in cellular functions such as inflammation, calcium (Ca^2+^) signaling, and response to oxidative stress after exposure times ranging from 6 hours to 1 year post-intoxication. However, there have been no studies to our knowledge examining the global gene expression response in non-cancerous and non-proliferating human neurons after BoNT/A exposure. In addition, it is not known whether previously observed physiological changes, such as neurite sprouting, are due to SNARE cleavage or to an alternative action of BoNT.

In this study, cDNA microarrays were used to analyze the effects of BoNT/A1 and a catalytically inactive BoNT/A1 derivative (BoNT/A *ad*) [18] on human neurons derived from human induced pluripotent stem cells (hiPSC). Our data indicate that the hiPSC-derived neurons used in this study and intoxicated with either BoNT/A or BoNT/A *ad* show few transcriptionally-linked changes after 2 days of intoxication. However, significant transcriptional changes were observed at 14 days post intoxication. Interestingly, gene expression changes were similar in both BoNT/A and BoNT/A *ad*-intoxicated cells, with strong differential changes in genes related to Ca^2+^ channel signaling and neurite sprouting, indicating that catalytic activity on SNAP-25 is not necessary for these changes to occur.

## Materials and Methods

### Botulinum neurotoxin

Isolated pure 150 kDa BoNT/A1 toxin heterodimer was obtained from the *Clostridium botulinum* strain Hall A*-hyper* as previously described [29]. Specific activity was determined by mouse bioassay (MBA) [30], [31] to be 1.25×10^8^ mouse LD_50_ Units/mg. Full-length BoNT/A *ad* (atoxic derivative), which had an over 100,000-fold reduced toxicity in mice compared to BoNT/A1 (9×10^2^ mouse LD_50_ Units/mg), was prepared as a recombinant protein expressed in insect cells via a baculovirus expression system as previously described [18].

### Neuronal cell culture

The neurons used for this study were cryopreserved hiPSC-derived neurons (iCell Neurons) purchased from Cellular Dynamics International (Madison, WI). The cell population was a 97% pure population consisting of GABAergic and glutamatergic neurons, with a small percentage of dopaminergic neurons (information provided by CDI, Madison, WI). Thawing and plating of the cells was performed as recommended by the manufacturer. Approximately 650,000 live cells were plated per well in a 6-well plate (Techno Plastic Products [TPP]) treated with 0.01% poly-L-ornithine (PLO) (Sigma) and coated with 8.3 µg/ml Matrigel (Biosciences). In order to ensure same cell populations throughout the experiment, the thawed vials of cells were pooled and mixed, before cells were counted and seeded for the entire study. Cells were maintained in media supplied by Cellular Dynamics in a 37°C, 5% CO_2_ incubator.

### Cell exposure to botulinum neurotoxin

Cells were exposed to 10 pM of BoNT/A or BoNT/A *ad* suspended in 3 ml of neuronal maintenance medium and incubated for 48 h. All cells were gently washed twice with 5 ml of fresh medium to remove extracellular toxin, and RNA was harvested as described below for the 2 d post exposure; the cells were maintained for another 12 d before harvesting RNA for the 14 d post exposure. All experiments were performed in triplicate. Non-exposed control samples were concurrently grown with exposed cells and harvested at the same time, and all mechanical manipulations including addition and removal of toxin to exposed cells was duplicated in the non-exposed samples with fresh medium not containing toxin. In parallel, two more triplicate sets of cells were exposed to the same toxin concentrations to confirm SNAP-25 cleavage. These samples were lysed in 1× lithium dodecyl sulfate (LDS) sample buffer (Invitrogen) and analyzed for SNAP-25 cleavage using Western immunoblotting as previously described [32], [33]. Briefly, analysis was performed via densitometry using a Foto/Analyst FX system and TotalLab Quant software (Fotodyne), correcting for background signal, with data plots being generated with Excel (Microsoft).

### Preparation of samples and microarray analysis

Total RNA was isolated from exposed and non-exposed iCell neurons at 2 d and 14 d post-initial exposure using an RNeasy mini kit (Qiagen) with addition of β-mercaptoethanol (βME) to the lysis buffer as recommended by the manufacturer. DNA was removed during isolation using an RNeasy RNase-free DNase kit (Qiagen); for samples analyzed by qPCR an additional DNase treatment was performed (Turbo DNase kit, Ambion). Samples were stored at −20°C after isolation.

RNA labeling and cDNA hybridization was performed by the University of Wisconsin-Madison Biotechnology Center Gene Expression Center (GEC, Madison, WI). After quantification (NanoDrop 2000) and determination of sample integrity (Agilent 2100 Bioanalyzer, RNA 6000 Pico chip), 400 ng of each sample was labeled with an Ambion MessageAmp Premier IVT kit. After cDNA fragmentation, 10 µg of each sample was hybridized to an individual Affymetrix HG U133 Plus 2.0 gene chip according to the methods described in the Affymetrix “Genechip Expression Analysis Technical Manual.” Samples were hybridized for 16 h at 45°C in an Affymetrix Hybridization Oven 640. Post-processing was conducted using an Affymetrix GeneChip Fluidics Station 450 following HG U133 Plus 2.0-specific protocols provided by Affymetrix, and each GeneChip was scanned using an Affymetrix GC3000 G7 scanner. Data was obtained from the scanned images using the Affymetrix Expression Console v 1.2.0.20 software.

Gene enrichment was conducted on all samples using GeneSpring 12.6 (Agilent) software. Robust Multi-array Analysis with correction for oligo GC content (GCRMA) was used for summarization with quantile normalization, and background reduction was performed on all samples by filtering for probes flagged as present by the MAS 5.0 algorithm. Normalized (log 2) data were then subjected to a two-way analysis of variance (ANOVA) with Benjamini Hochberg FDR multiple testing correction and filtered for primary interactions (time, exposure) with P-values <0.05. Relevant interactions were used in determining genes meeting fold expression criteria among the various conditions, with an expression change greater than 2.0 being used to determine significance. Entities with an Affymetrix probe ID but no official gene symbol or designated title were removed from analysis and not counted in the reported data. Analysis of gene enrichment was performed using DAVID Bioinformatics Resources [34], [35], using the GOTERM_BP_FAT, GOTERM_CC_FAT, and GOTERM_MF_FAT datasets. The data discussed in this publication have been deposited in NCBI's Gene Expression Omnibus [36], [37] and are accessible through GEO Series accession number GSE58149.

### Validation by real-time quantitative PCR analysis

In order to confirm the expression values from the microarray analysis, qPCR was conducted with all 18 samples used for microarray analysis. Primers were designed using the web-based Primer-BLAST tool (NCBI) [38]. Primers were tested for correct size and amplification of a single product by PCR using non-exposed iCell Neuron RNA as a template, followed by visual confirmation of product using gel electrophoresis (2% agarose gel). Reverse transcription of RNA into cDNA was done using Superscript II (Life Technologies) according to the manufacturer's instructions. Samples were diluted 1∶10 and efficiency tests were carried out on primers using a temperature gradient and DNA melt curve analysis; primers, product lengths, and efficiency values are given in Table 1. H2AFY and RTN4 were chosen as optimal reference genes based upon geNorm algorithm computation (qbase + software, Biogazelle).

**Table 1:** List of all primer sequences used for qPCR.

Gene	Forward Primer	Reverse Primer	Product Length (bp)	Efficiency (E = 10^(−1/slope)^-1)
CHRM3	TTGGCATCACCAAGCACCTA	GTGGAGCTTTGTTCAGTTCCC	116	109.4%
DAPK3	GGCCCCAGAGATTGTGAACT	TTCACGGCTGAGATGTTGGT	147	97.2%
H2AFY	GAGGTGGGACCCCTTTCATTT	GACACGGTCTGGAACACAGT	151	91.8%
MLL set #1	GACGTTGGCTTTGCATCTGG	AGAGGGCTCCTTTTGTGCAT	110	102.2%
MLL set #2	AGATGAAGAAGTCAGAGTGCGA	GAGCCACTTCTAGGTCTCCC	90	103%
NRGN	CCTACATCTCCGTGCCACTC	AGGGACTTCTCTTCCGGTCA	123	111.8%
RDH10	GCTGCATACAGAAAGTGCCC	TTGCAGAGCTGCTATGTATTCT	92	115%
RTN4	CCACCCCACAGTGCTTGATA	TTGGAGTTCTATGTGTGTGGCA	142	94.9%

QPCR was performed with a Roche LightCycler 480 in triplicate with SYBR Green fluorescent dye (SsoAdvanced SYBR Green, Bio-Rad) in triplicate according to the Roche LightCycler 480 SYBR Green I Master protocol. Fold change was determined using qbase+.

## Results

### BoNT/A1 but not BoNT/A *ad* exposure of hiPSC derived neurons resulted in SNAP-25 cleavage

**Figure 1:**
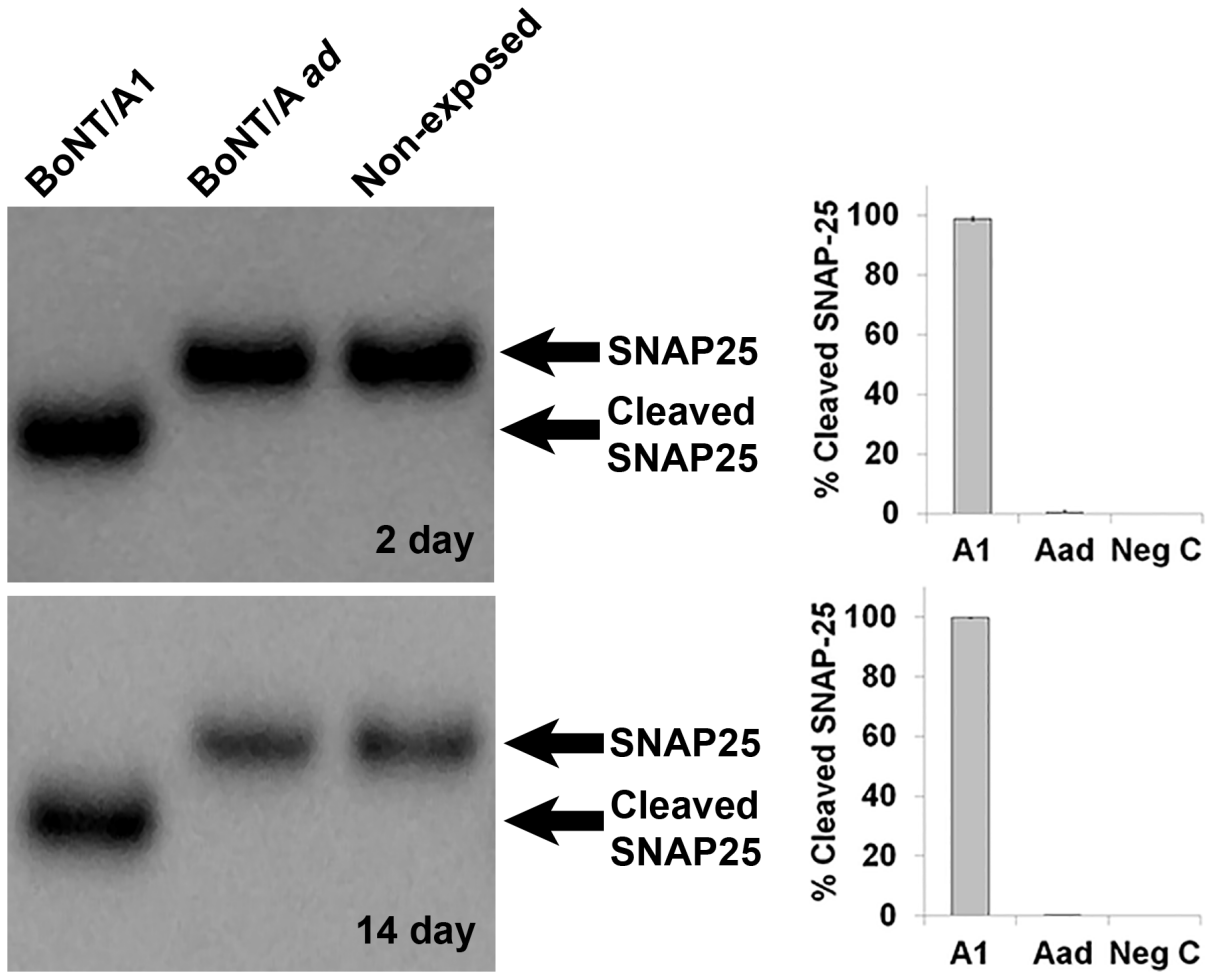
Confirmation of BoNT activity in exposed and non-exposed iCell Neuron samples by Western blot. A representative Western blot is shown for both 2 days and 14 days exposure. Percentage of SNAP-25 cleavage, conducted via densitometry and averaged for all three replicates after correcting for background values, is represented as a bar graph; error bars represent standard deviation of the three replicates.

The sensitivity of iCell Neurons to BoNT/A1 has previously been established [39]. In order to confirm catalytic activity of BoNT/A1 and absence of catalytic activity of BoNT/A *ad* in the cells at the concentrations used for the microarray analysis, iCell Neurons were exposed to 10 pM BoNT/A1 or BoNT/A *ad* for 48 h. Toxin was removed by washing, and at day 2 (immediately after 48 h exposure) and day 14 cells were lysed and SNAP-25 cleavage was determined by Western immunoblot. As expected, exposure of the neurons to BoNT/A1 resulted in detection of fully cleaved SNAP-25, while only uncleaved SNAP-25 was visible in both the BoNT/A *ad* exposed cells or the non-exposed control cells (Fig. 1).

### There were minimal differences in expression profiles of neurons exposed to BoNT/A1 and BoNT/A *ad* for 2 days

Comparison of gene expression profiles of BoNT/A1 and BoNT/A *ad*-exposed cells at 2 d post-initial exposure against their accompanying non-exposed cells, which had undergone all of the same manipulation steps as the exposed cells but without toxin added, revealed only minimal differential gene expression profiles. There were no genes differentially regulated greater than 2-fold between the two exposed cell populations, and only 6 total genes were differentially regulated more than 2-fold in BoNT/A1 or BoNT/A *ad*-exposed cells compared to non-exposed cells (Table 2).

**Table 2:** List of genes differentially regulated greater than 2-fold at 2 days post toxin exposure.

Population Comparison	Genes	Fold Change Microarray (qPCR)
BoNT/A1 vs BoNT/A *ad-*exposed	None	NA
BoNT/A1 vs Non-exposed	DAPK3	−2.4 (1.0)
	CNKSR2	−2.3
	FOS	−2.2
	SCYL2	−2.2
	RC3H2	−2.1
BoNT/A *ad* vs Non-exposed	MLL	−2.3 (1.0)

### The general expression profile was significantly altered after 14 days post exposure in both BoNT/A1 and BoNT/A *ad*-exposed neurons

Comparison of the expression profile of non-exposed iCell Neurons at 2 d and 14 d revealed no differentially expressed genes (P<0.05). This suggests that these hiPSC-derived neurons are transcriptionally stable over the course of 14 d in culture, which is similar to data received from an expression study by the producer of cells (CDI, personal communication).

In contrast, at 14 d post-BoNT/A1 and BoNT/A *ad* exposures, significant changes were detected in the expression profiles of the exposed neurons as compared to non-exposed neurons. Of 3320 genes correlated with exposure (P<0.05), 354 and 503 genes were differentially regulated 2-fold or greater in BoNT/A1 and BoNT/A *ad*-exposed cells compared to non-exposed populations. However, the vast majority of these genes showed only minor differences in expression levels (below 5 fold), and only ∼12% were differentially regulated more than 5-fold. Surprisingly, the most highly regulated genes showed increased expression in BoNT/A1 and BoNT/A *ad*-exposed neurons (Table 3, Table S1). This was unexpected because the BoNT/A *ad* did not cleave SNAP-25 in the conditions used in this assay.

**Table 3:** List of select genes differentially regulated at 2 weeks post-exposure, with direction in relation to non-exposed populations.

Gene Name, Entrez Gene #	Full Name	Function in Neurons	Regulation^a^	qPCR confirmed	References
Neurite Outgrowth					
FGFR2, 2263	Fibroblast growth factor receptor 2	-Involved in the development of frontal brain regions -Regulates Neurogenesis in human endometrial stem cells	21.6, 26.2	NA	[56], [57]
RDH10, 157506	Retinol dehydrogenase 10	-Involved in synthesis of retinoic acid in humans -Retinoic acid controls generation of neocortical neurons during embryonic life in mice	19.1, 21.0	11.2, 11.6	[58], [59]
ADAMTS3, 9508	Metallopeptidase with thrombospondin type 1 motif, 3	-Constitute a family of zinc metalloproteinases which target and process extracellular matrix proteins. -May be a collagen processing enzyme	14.2, 13.2	NA	[60]
SPARCL1, 8404	SPARC-like 1 (hevin)	-Involved in the adhesion between neurons and radial glial cells -influence the function of astroglial cells in CNS	13.4, 14.6	NA	[61]
SALL3, 27164	Spalt-like transcription factor 3	-Required for the terminal maturation of neurons destined for the glomerular layer -Positively regulate tyrosine hydroxylase expression	12.5, 13.6	NA	[62]
SPON1, 10418	Spondin 1, extracellular matrix protein	-Positively counteract neurite retraction -Implicated in cell-matrix and cell-cell adhesion and plays an important role in axonal pathfinding	9.5, 9.5	NA	[63], [64]
NEGR1, 257194	neuronal growth regulator 1	-Involved in the in the pathogenesis of neuroblastoma (cancer that develops from immature nerve cells) -Exogenous expression in neuroblastoma cells induced significant inhibition of cell growth	−1.5, −5.3	NA	[43], [65]
Ca^2+^ Channel Sensitization					
CACNA2D3, 55799	Calcium channel, voltage-dependent, alpha 2/delta subunit 3	-Identified as pain genes, important for heat pain sensitivity -Encodes α_2_δ subunits that is involved in synaptogenesis	50.6, 48.6	NA	[66], [67]
CHRM3, 1131	Cholinergic receptor, muscarinic 3	-Member in muscarinic acetylcholine receptor family -Reduces axial elongation	9.0, 9.7	3.9, 4.1	[68], [69]
NRGN, 4900	Neurogranin (protein kinase C substrate, RC3)	-Main postsynaptic protein regulating the availability of calmodulin-Ca^2+^ in neurons -Expressed exclusively in the brain, particularly in dendritic spines	14.8, 17.8	6.1, 6.5	[70]

a The first number represents regulation of BoNT/A1 in relation to non-exposed samples, the second number represents regulation of BoNT/A *ad* in relation to non-exposed samples.

**Figure 2:**
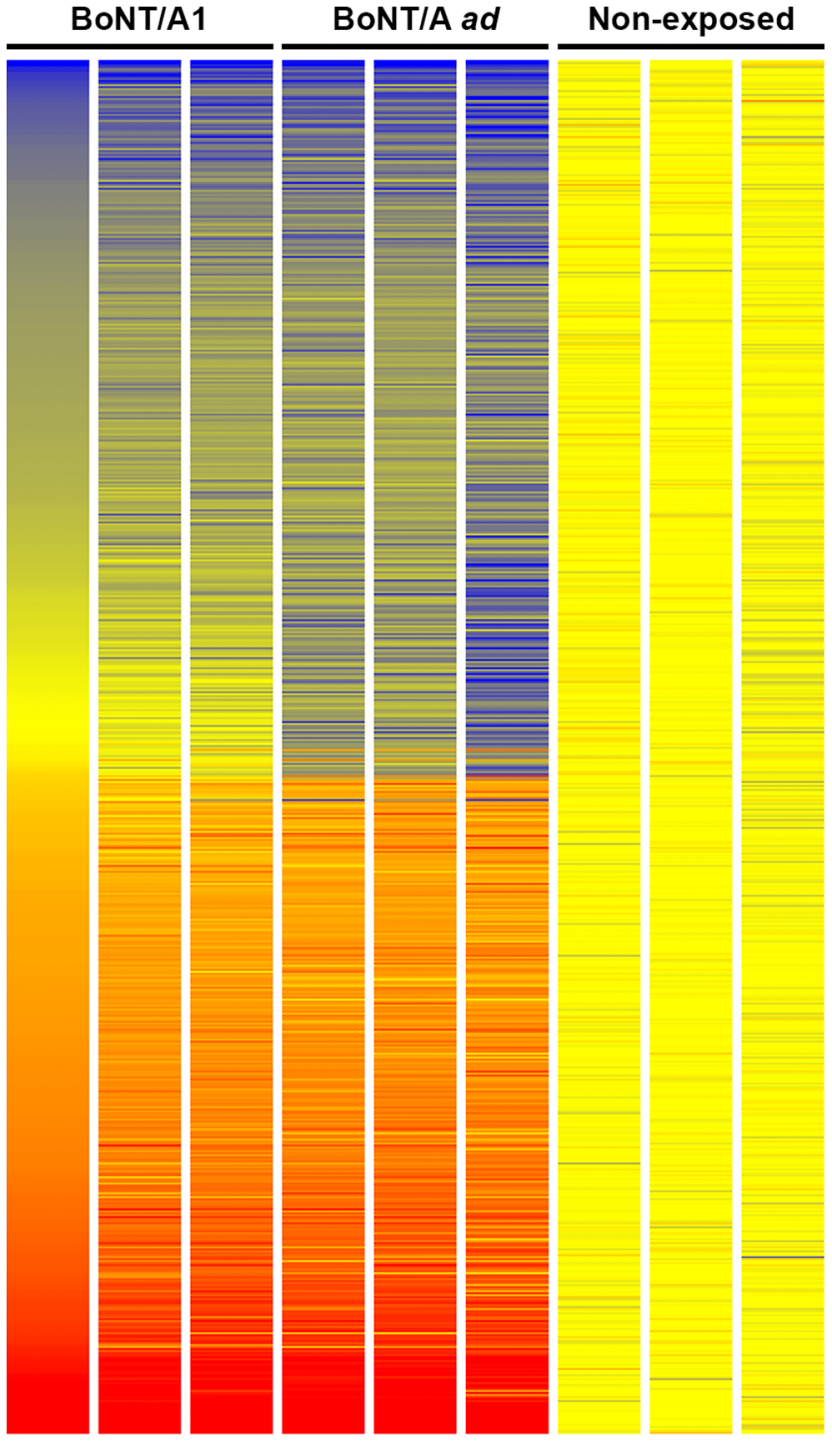
Heat map comparison of significantly regulated genes after 2 weeks of exposure to BoNT/A1 and BoNT/A *ad*. All genes differentially expressed greater than 2-fold in either BoNT/A1 or BoNT/A *ad*-exposed cells are represented, sorted by normalized expression level as determined by the first replicate of BoNT/A1-exposed cells. All three replicates of each condition are shown as individual columns. Blue represents lower expression, while red represents higher expression on a scale of −3.0 to 3.0.

Most notably, the highly upregulated genes in both BoNT/A1 and BoNT/A *ad*-exposed neurons encoded proteins involved in neurite outgrowth (FGFR2, RDH10, ADAMTS3, SPARCL1, SALL3, SPON1) and calcium (Ca^2+^) channel activity (CACNA2D3, CHRM3, NRGN). In addition, several other genes were significantly upregulated, including genes associated with neuronal survival (PDE5A) and certain classes of migraine headaches (ATP1A2). Table 3 lists the most significantly expressed genes and their function. Of the genes that were significantly downregulated many were associated with cellular scaffolding and matrices, important processes involved in cellular proliferation, spatial organization, and adhesion. Examples include *COL1A1* [40],*COL8A1* [41], and *COL11A1* [42], genes encoding subunits of types I, VIII, and XI collagen. Functional annotation clustering analysis of differentially expressed (2-fold or greater) genes in BoNT/A1 and BoNT/A *ad*-exposed populations at day 14 compared to non-exposed cells using DAVID revealed significant enrichment of genes involved in cell motion, adhesion, and signaling, as well as specific neuron differentiation, development, and projection (Table S2). The differential expression of several genes (*CHRM3*, *DAPK3*, *MLL*, *NRGN*, *RDH10*, and *RTN4* and *H2AFY* as reference genes) was confirmed by qPCR (Tables 2 and 3). Overall, the qPCR data indicated an ∼2-fold lower differential gene regulation than indicated by microarray, which suggests that changes detected by microarray that are below 2-fold or even 3-fold may be less significant. There were 36 and 190 genes that are regulated greater than 2-fold exclusively when comparing BoNT/A1 BoNT/A *ad* to non-exposed cells, respectively. However, many of these genes were still regulated similarly in BoNT/A1 and BoNT/A *ad* exposed neurons, which are visualized generally (Fig 2) and in greater detail (Table S1). Direct comparison of genes differentially regulated in neurons exposed to BoNT/A1 or BoNT/A *ad*-exposed at 14 days post exposure showed only 60 genes differentially expressed greater than 2-fold (Table 4, Table S3). The most significant difference was observed for NEGR1, a neuronal growth regulator [43] that was upregulated 3.5-fold by BoNT/A1 compared to BoNT/A *ad*. Interestingly, when differential expression of NEGR1 in BoNT/A1 or BoNT/A *ad* exposed neurons was compared to non-exposed neurons, NEGR1 was downregulated significantly in BoNT/A *ad*-exposed cells, but not in BoNT/A1-exposed cells (-1.6-fold), thus indicating that this gene is not significantly regulated in BoNT/A1-exposed neurons. HSPA6, a heat shock/general stress protein [44], was the only gene that is downregulated (-2.0-fold) in BoNT/A1-exposed cells compared to BoNT/A *ad*-exposed cells.

**Table 4:** Number of genes differentially regulated between BoNT/A1 and BoNT/A *ad*-exposed cells at 14 days post toxin exposure.

Fold Change Range	Number of Genes Differentially Regulated
3.5+	1
3.0–3.5	6
2.5–3.0	9
2.0–2.5	44

When two differentially regulated entities were identified as the same gene, the average of the differential expression was used for categorization.

## Discussion

BoNT/A1 is widely used as a pharmaceutical in medicine, with more than 3.5 million uses in the US in 2013 alone [45]. One of the hallmarks of these toxins is their long duration of action, lasting for 3–6 months post local injection in humans [2]. In a recent paper we described that this long duration of action was partly due to a persistence of active BoNT/A LC inside the neurons [14], which supported the results of Montecucco's group in seminal studies [12]. However, despite their widespread use and persistence in neurons, little is known of the transcriptional changes that take place in intoxicated neurons. In this report, we describe RNA expression profiles in neurons exposed to BoNT/A1 or to the recombinant BoNT/A1 derivative (BoNT/A *ad*) which did not show SNAP-25 cleavage in the assay used here. BoNT/A *ad* is being studied as a delivery platform to target drugs specifically to neuronal cells [18], and results have indicated that it can enter neurons in an analogous mechanism to BoNT/A1 [19].

In this study we showed that there were only minor changes in expression profiles of the neurons exposed to BoNT/A1 or BoNT/A *ad* compared to non-exposed neurons at 2 days post toxin addition, while there were significant changes in expression profiles after 2 weeks. Interestingly, the more highly regulated genes (FC>5.0) were similarly regulated in BoNT/A1 and BoNT/A *ad* in comparison to non-exposed cells (Table 3). This suggests abundant transcriptional changes in neurons, independent of SNAP-25 cleavage following BoNT exposure. BoNT/A *ad* has been shown to be at least 100,000-fold less toxic in mice than BoNT/A1 [19] and does not cleave SNAP-25 in neurons at the concentrations used in this study, and its effects on transcriptional changes are due to unknown mechanisms. Since BoNT/A *ad* binds to SNAP-25, it is possible that the observed effects are due to an interruption of intracellular signaling by SNAP-25 that is independent of catalytic cleavage, which may be reflected in changes to Ca^2+^ channel activity [46]. It has previously been observed that neuritogenesis in response to BoNT/A1 intoxication in cultured mouse spinal cord cells is dependent on the binding of the toxin to the neuronal membrane and independent of SNARE cleavage [20]. The majority of highly regulated genes in BoNT/A1 and BoNT/A *ad*-exposed cells compared to non-exposed cells were involved in neuritogenesis, supporting that the transcriptional changes are independent of SNAP-25 cleavage. The observation of distinct Ca^2+^ channel-related genes undergoing upregulation in human neurons upon BoNT exposure appears to be novel. However, the hypothesis that changes in SNAP-25 activity affects Ca^2+^ channel activity, particularly among astrocyte signaling, has been observed in mouse hippocampal neurons [46]. Furthermore, the inhibitory effect of SNAP-25 on Ca^2+^ channel activity in mouse hippocampal neurons has been reported [47]. The direct effect of BoNT intoxication on Ca^2+^ channel signaling indicates both an increase [48] and a decrease in Ca^2+^ utilization [49]. Future studies are required to further investigate these observations on a functional level and to determine whether association or cleavage of SNAP-25 or another property of BoNT/A1 and BoNT/A *ad* is responsible for the observed transcriptional changes. Finally, it is unlikely that the transcriptional changes were caused by an indirect effect of a contaminant common to both, BoNT/A1 and BoNT/A *ad* is unlikely, as the two proteins were produced and purified by completely independent procedures. The 150 kDa BoNT/A1 was purified from *C. botulinum* by published biochemical methods [29], whereas the BoNT/A ad was expressed in and purified from insect cells via a his- and strep-tag [18].

The neuronal cell population used in this study is a comprised primarily of GABAergic and glutamatergic neurons, resembling human forebrain neurons. This system differs from another microarray study that investigated gene expression in both a continuous cell line and epithelial cells [26]. Differences between the results of the two studies may be attributed to the different cell models, as well as differences in exposure parameters to BoNT/A1, including exposure time, toxin concentrations, and the use of BoNT/A1 complex compared to purified neurotoxin. Irrespective of the differences in methodologies, both studies demonstrated upregulation of genes involved in neurite outgrowth. While our cell model provides a human non-cancerous neuronal population, it does not encompass other cell types that are also components of the central nervous system (CNS). Microglial cells, for instance, form a reactive protection network that protects the CNS against harmful stimuli [50]. These cells not only react to environmental stimuli, but also communicate with neuronal cells and exert influence on neuron function [51]. Astrocytes together with neuronal pre- and post-synaptic terminals make up the tripartite synapse in the CNS [52], and these also respond to environmental stimuli and interact with neurons by Ca^2+^ channel variations [53]. Signals can be transferred along astrocyte chains, delivering messages to and from active and inactive synapses [53]–[55]. The absence of these interacting cells in the neuronal population used in this study may affect the overall transcriptional response of BoNT-exposed neurons. In addition, other neuronal subpopulations, such as motor neurons or nociceptors may have specific responses to BoNTs that could not be revealed in this study.

In summary, these data show that intoxication by BoNT/A1 and BoNT/A *ad* elicit similar responses in human neurons, particularly in genes that are related to neuritogenesis and Ca^2+^ channel sensitization. These findings suggest that cleavage of SNAP25 is not necessarily the key process that leads to the transcriptomic changes observed. It is unknown whether cellular entry or physical binding of the toxins to neurons are responsible for the observations described here. Further transcription-based research on the effects of BoNTs as well as their complexing proteins in different cell models is required to determine the mechanisms affecting gene expression. BoNT/A *ad* has previously been suggested as a general delivery vehicle to target compounds to neurons [18], and the similarities in genes differentially regulated in response to both BoNT/A1 and BoNT/A *ad* support such use.

## Supporting Information

Table S1 **List of all named genes correlated with exposure at 14 d post-exposure with a differential expression greater than 2-fold in either BoNT/A1 or BoNT/A** ***ad*** **-exposed cells when compared to unexposed cells.** The data are represented by two worksheets; the first is sorted by differential expression as determined by BoNT/A1 expression compared to control, and the second is sorted alphabetically by gene symbol. Duplicate entires (distinct probes that target identical genes) are grouped together by outline.
(XLSX)

Table S2 **Functional Annotation Clustering output on genes that are differentially expressed in either 14 d BoNT/A1 or BoNT/A** ***ad*** **-exposed cells compared to non-exposed cells.**
(XLSX)

Table S3 **List of all named genes correlated with exposure at 14 d post-exposure with a differential expression greater than 2-fold in BoNT/A1-exposed cells when compared to BoNT/A** ***ad*** **-exposed cells.**
(XLSX)
